# What can body ownership illusions tell us about minimal phenomenal selfhood?

**DOI:** 10.3389/fnhum.2014.00946

**Published:** 2014-11-24

**Authors:** Jakub Limanowski

**Affiliations:** ^1^Berlin School of Mind and Brain, Humboldt-Universität zu BerlinBerlin, Germany; ^2^Neurocomputation and Neuroimaging Unit, Department of Education and Psychology, Freie Universität BerlinBerlin, Germany

**Keywords:** body ownership, Illusion, self, minimal self, rubber hand illusion

Illusions have become an invaluable tool for investigating how the sense of a body as *one's own* is constructed and maintained: During the *rubber hand illusion* (RHI, Botvinick and Cohen, 1998), congruent touch to one's hidden hand and a fake counterpart produces an illusion of feeling the touch on the fake hand and, more strikingly, an experience of the fake hand as part of one's body (Ehrsson et al., 2004; Tsakiris, 2010). The principles of the RHI paradigm have been extended to various body parts (Petkova et al., 2011), including even the face (Tsakiris, 2008; Apps et al., 2013); most notably, the RHI has been induced for the entire body *(full body illusion, FBI)*, producing similar behavioral and neural responses (anxiety responses, ownership of the fake body, misperception of one's physical location; Ehrsson, 2007; Lenggenhager et al., 2007; Maselli and Slater, 2013). Such *body ownership illusions (BOIs)* have generated a substantial amount of research and invaluable insights into the mechanisms of body ownership (Tsakiris, 2010; Blanke, 2012; Moseley et al., 2012). The special importance of these illusions lies in the fact that what they manipulate—the sense of having a body—is one of the enabling conditions of *minimal phenomenal selfhood* (MPS, Gallagher, 2000; Blanke and Metzinger, 2009; Metzinger, 2013a). MPS is defined as the most basic possible kind of self-consciousness or self-awareness (Blanke and Metzinger, 2009; Gallagher and Zahavi, 2010), and investigating its enabling conditions may help us understand what it takes for an organism to have the experience of being a self. Nevertheless, in this paper I will argue that it is still unclear what exactly the mechanisms revealed by BOIs tell us about MPS, and that this needs to be clarified via a joint effort of phenomenological analysis and formal accounts of self-modeling.

BOIs rest on the induction of some crossmodal conflict (e.g., touch seen on a fake hand but felt on one's real hand), which violates the predictions of one's body-model about the unity of one's body (Hohwy, 2007). This conflict is resolved by the brain by remapping modality-specific body part-centered reference frames onto each other (e.g., proprioception onto vision), so that the multimodal representation of the body and the space surrounding it remains coherent (Holmes and Spence, 2004; Makin et al., 2008; Tsakiris, 2010; Blanke, 2012). Thereby the spatio-temporal and anatomical constraints of BOIs (touch needs to occur simultaneously at corresponding locations and on a congruent body part in an anatomically plausible posture) suggest that multisensory input has to be compatible with a prior representation of the body (Tsakiris and Haggard, 2005; De Preester and Tsakiris, 2009; Tsakiris, 2010; Moseley et al., 2012). The brain seems to make a probabilistic either-or decision based on current sensory input under a prior body model, which during BOIs results in the replacement of the real body part by the “owned” fake body part (Longo et al., 2008; Moseley et al., 2012). And indeed, the neural mechanisms integrating multisensory information during the RHI may be similarly employed for one's real body parts (Gentile et al., 2013). When the rubber hand is threatened or injured, participants show behavioral, and neural anxiety responses similar as for one's real body part (Ehrsson et al., 2007). BOIs may even affect the regulation of one's physiological states: During illusory ownership, one's real limb's temperature may be downregulated (Moseley et al., 2008), and even the immune system may decrease “protection” of the own limb (Barnsley et al., 2011; Costantini, 2014). In sum, there is compelling evidence that BOIs interfere with the representation of one's body. Upon closer inspection, however, the fact that BOIs isolate “the various components that converge in the holistic experience of our bodies” (Maselli and Slater, 2013) may be a fundamental limitation when it comes to relating them to MPS.

## Manipulating (features of) the self: understanding the implications of body ownership illusions for minimal phenomenal selfhood

Phenomenological analysis has emphasized the paradox role of the body in our experience (Legrand, 2010) as both an “objective” body that is a physical thing and thus part of the world, and a “subjective” or “lived” body that is our means of experiencing and interacting with the world (this can be traced back to Husserl, Merleau-Ponty, and arguably Sartre, see Gallagher, 1986; Gallagher and Zahavi, 2010). Crucially, during interaction with the world, we usually do not experience the lived body as a thing enabling this interaction, and it may be this very “experiential absence” of the body that gives us the feeling of “being there” in the world (Gallagher, 1986; Metzinger, 2004). Such a phenomenological distinction may be of particular relevance for BOIs, which isolate and manipulate, and direct attention to perceptual features of oneself such as visual appearance or physical location. In William James' words, BOIs may interfere with the “me” (i.e., the features that one ascribes to oneself) but not the “I” (the subject of experience) (James, 1890; Christoff et al., 2011). Thus, the paradox role of the body may be part of what makes the interpretation of BOIs and their relation to MPS so difficult: When we speak of body “ownership” in these paradigms, we may only refer to self-identification with the perceived body.

Although MPS can be broken down into distinct features (e.g., self-identification, self-location in space and time, or a first-person perspective), it is still one phenomenal property that “does not have proper parts that could themselves count as a kind or type of self-consciousness” (Metzinger, 2013a, 2004). MPS thus acknowledges the “global and unitary character of self-consciousness” (Blanke and Metzinger, 2009). This unified character of MPS—characterized by the fact that we cannot introspectively access its underlying mechanisms and components—is what lends our experience its phenomenal “realness” (Metzinger, 2004, 2013a). As we have recently argued (Limanowski and Blankenburg, 2013), this MPS conceptualization is elegantly compatible with a theory of cortical information processing based on predictive coding (Friston, 2010): The *free energy principle (FEP)* postulates that the brain implements a hierarchical generative model of the world (including the organism itself) that constantly predicts the causes of its sensory input, and that may be updated if its predictions fail. Due to the model's constant bidirectional and hierarchical information flow with the aim of reducing overall prediction error, this model is spatially, temporally, and phenomenally centered onto the organism itself (Hohwy, 2007, 2013; Friston, 2011; Limanowski and Blankenburg, 2013; Apps and Tsakiris, 2014). The FEP account, albeit mechanistic in nature, thus acknowledges that one's self-representation is plastic (i.e., probabilistic), hierarchical, and yet unified. It builds upon existing neuropsychological models of body ownership (e.g., Makin et al., 2008; Tsakiris, 2010), and shares key assumptions about being a self with the MPS conceptualization—most importantly, that the phenomenal self is a result of probabilistic self-modeling.

Thereby the MPS and FEP accounts both emphasize that such probabilistic self-modeling is a “risky business” for the phenomenal self: When the current generative model is abandoned in favor of another model that fits the sensory data better, the agent in its present form—encoded as the model evidence of the self-centered world-model—ceases to exist (Friston, 2011). Luckily, this does not need to happen: If prediction error can be explained away at lower levels, there is no need to adjust higher-level predictions, let alone to abandon the current world-model. In the case of BOIs this means that if the probabilistic representation of one's physical features can be updated to eliminate the surprise originating from the ambiguous sensory input, there is no need to abandon the actual self-model (Hohwy, 2013; Limanowski and Blankenburg, 2013; Apps and Tsakiris, 2014). Such an updating of self-representations is demonstrated, for example, by the increased perceived similarity of the “owned” dummy hand (Longo et al., 2009), and even of another face (Tsakiris, 2008; Apps et al., 2013) to oneself, or by the fact that the mere expectation of an upcoming touch on the dummy hand may evoke a BOI (Ferri et al., 2013).

In this light, BOIs can be conceived of as targeting specific inferential mechanism employed by the organism, thus directly confirming the functional architecture suggested by probabilistic models of MPS—but this also opens up some questions. It seems that BOIs primarily affect mechanisms operating at lower levels of self-modeling, because the induced conflict can readily be resolved without inducing panic or pathological conditions, and because the recalibration of one modality onto another relies on relatively basic multisensory mechanisms (Tsakiris, 2010; Blanke, 2012; Gentile et al., 2013). On the other hand, the adjustment of physiological responses (Ehrsson et al., 2007; Moseley et al., 2008; Barnsley et al., 2011) might imply that BOIs also affect higher levels of the self-model (Seth, 2013). To understand the implications of BOIs for MPS, it is crucial to identify which levels of MPS they affect: When do these illusions merely alter perceptual features of oneself, and when—if at all—do they in fact affect MPS *per se*?

Metzinger (2013a,b) has recently clarified the MPS conceptualization by introducing the term *phenomenal unit of identification* (UI), “the phenomenal property with which we currently *identify*, exactly the form of currently active conscious content that generates the subjective experience of ‘I *am* this”’ (2013b). As I take it, the UI concept can be used in two ways: It specifies the content with which the self-model identifies—this self-identification leads to the experience of MPS (Metzinger, 2004). Formally, we may think of the UI as the evidence or the “origin” of the current generative model (the “region of maximal invariance,” Metzinger, 2013a; see also Friston, 2011; Limanowski and Blankenburg, 2013).

The important point is that the UI concept may help to answer our question: BOIs *do* affect MPS, just not in its entirety. The resolution of BOI-evoked conflicts by changing the representation of certain features of oneself, despite attacking fundamental conditions of MPS, may not be sufficient to change the UI. Even the extension of BOIs to the fully body—although surely a step in the right direction—does not necessarily imply a change in the UI: For example, since FBIs seem to employ the same multisensory mechanisms as the RHI (Petkova et al., 2011; Maselli and Slater, 2013), it could be that what becomes subject to the illusion is actually not the whole body, but just the stimulated part of the body (e.g., the torso in the FBI, Smith, 2010; Metzinger, 2013a; see Tsakiris et al., 2006, for such evidence). More importantly, FBIs may also manipulate only individual features of one's self-representation such as one's visual appearance or physical location.

Interestingly, however, Metzinger (2013a,b) proposes that the UI may change during mind wandering episodes, bodiless dreams, and some forms of out-of-body experiences. The resulting claim that an explicit body representation may actually not be required for MPS (Metzinger, ibd.) is in line with the proposal that a core self is present even when disconnected from exteroceptive sensory input (e.g., during sleep, Park and Tallon-Baudry, 2014). Such proposals are inspiring, but they also show that much work still needs to be done to understand the exact relation between the body, MPS, and its UI. Probabilistic models following the FEP offer a promising way of formally describing the underlying self-modeling mechanisms, but in addition, new experimental approaches addressing specific levels of MPS are needed. To avoid confusion, these approaches should acknowledge the dual role of the body as both subject and object of experience.

## Conclusion

BOIs have proven an important tool for understanding MPS. But one should keep in mind that BOIs manipulate individual features of one's self-representation, and that this manipulation of certain dimensions of MPS not necessarily affects the UI. Nevertheless, if situated within a phenomenologically inspired probabilistic model, BOIs can contribute to our understanding of MPS by clarifying its hierarchy, and to further developing the UI concept and its relation to the body. Whether we can develop paradigms that manipulate the UI to actually create the illusion of *being someone else* is a different question, but there are promising new developments that encourage to pursue it.

### Conflict of interest statement

The author declares that the research was conducted in the absence of any commercial or financial relationships that could be construed as a potential conflict of interest.
